# Causal associations of COVID‐19 on neurosurgical diseases risk: a Mendelian randomization study

**DOI:** 10.1186/s40246-024-00575-y

**Published:** 2024-02-05

**Authors:** Lirui Dai, Liang Lyu, Peizhi Zhou, Shu Jiang

**Affiliations:** Department of Neurosurgery, Pituitary Adenoma Multidisciplinary Center, West China Hospital of Sichuan University, Chengdu, Sichuan China

**Keywords:** Neurosurgical disorders, COVID‐19, Genetic variants, Genome‐wide association study, Mendelian randomization

## Abstract

Many researchers have explored the potential association between one neurosurgical disease and coronavirus disease 2019 (COVID-19), but few systematically analyzed the association and causality between COVID-19 and various neurosurgical diseases. A Mendelian randomization analysis was conducted to evaluate the causal association between COVID-19 (including critically ill COVID‐19, hospitalized COVID‐19, and respiratory syndrome coronavirus 2 (SARS‐CoV‐2) infection) and 30 neurosurgical diseases within European populations. The consequences of inverse variance weighted models suggest that genetic susceptibility of critically ill COVID-19 may increase the risk of cerebral infarction (odds ratio [OR] = 1.02; *p*‐value = 0.006), genetic susceptibility of SARS-CoV-2 infection may increase the risk of stroke (OR = 1.02; *p*‐value = 0.047), and conversely, genetic susceptibility of hospitalized COVID-19 may reduce the risk of pituitary adenoma and craniopharyngioma (OR = 0.90; *p*‐value = 0.032). In addition, evidences revealed potential associations between genetic susceptibility of COVID-19 and spinal stenosis (OR = 1.03; *p*‐value = 0.028), diffuse brain injury (OR = 1.21; *p*‐value = 0.040) and focal brain injury (OR = 1.12; *p*‐value = 0.040). By testing for heterogeneity and pleiotropy, the above causal conclusions are robust. In summary, our analysis shows that COVID-19 has an independent and powerful causal influence on multiple neurosurgical disorders.

**Supplementary Information:**

The online version contains supplementary material available at 10.1186/s40246-024-00575-y.

## Introduction

Since 2019, the worldwide outbreak of the coronavirus disease 2019 (COVID-19), contributed by the severe acute respiratory syndrome coronavirus 2 (SARS-CoV-2) [1], has been rapidly disseminating, leading to an escalating tally of confirmed cases and fatalities, thereby presenting a grave menace to the well-being of the general populace. Many susceptibility factors and protective factors are closely associated with the infection or severity of the COVID-19 [2, 3]. There are also many COVID-19 patients who gradually recover from acute infection and develop post-COVID-19 syndrome, among which the invasion and influence on the nervous system are relatively common [4, 5]. Therefore, this paper mainly explores the correlation between genetic susceptibility to COVID-19 and neurosurgical diseases.

The effect of patients with post-COVID-19 syndrome on central nervous system diseases may affect the progression of neurological diseases by affecting the metabolism of neurons or glial cells [6, 7]. The nervous system is the most complex part of the body, and when the disease of the nervous system encounters COVID-19, the diagnosis, treatment and prognosis of patients will face a severe test. For many neurosurgical diseases, the duration of treatment has been significantly reduced due to better maintenance of health services during the COVID-19 pandemic, and life care has been improved for critically ill patients and those requiring recovery [8], but the impact of COVID-19 on overall survival has varied. Some patients did not have a significant influence on their survival and prognosis due to the COVID-19 pandemic [9], but it has also been reported that COVID-19 infection is a risk factor for poor prognosis of some craniocerebral diseases [10, 11]. Most of the above data are based on individual cases to broadly analyze the influence of COVID-19 on neurosurgical diseases, which is neither universal nor comprehensive. Therefore, systematic analysis of the influence of COVID-19 on neurosurgical diseases is currently a very important and meaningful study. This will be important for the health management and prevention of patients with these diseases as COVID-19 approaches.

Mendelian randomization (MR) studies employ genetic variation as instrumental variables to establish causal links between genetically determined exposures and disease. By mitigating conventional confounding and reverse causation, MR analysis has gained considerable traction in contemporary research to investigate the association between pertinent attributes and diseases. Numerous studies have systematically unveiled causal connections between COVID-19 and diverse cancers [12], as well as associations between COVID-19 and chronic ailments [13, 14]. However, the causal relationship between COVID-19 and neurosurgical disorders is still unclear, and exploring their correlation could help improve the management and treatment of neurosurgical diseases in the context of COVID-19 infection.

## Methods

### Study design

We employed a two-sample Mendelian randomization (MR) analysis to examine the causal association between COVID-19 and neurosurgical diseases. In MR researches, genetic variation serves as the most effective instrumental variable (IV). To mitigate potential biases, we derived IVs by adhering to three specific criteria. [15, 16].

1. The association hypothesis suggests that IVs should be closely associated with exposure levels;
2. The independence hypothesis indicates that IVs is not associated with any hidden confounding factors;
3. The exclusivity hypothesis states that genetic variation cannot be directly related to the consequence, but can only affect the consequence via exposure.

**Fig. 1:**
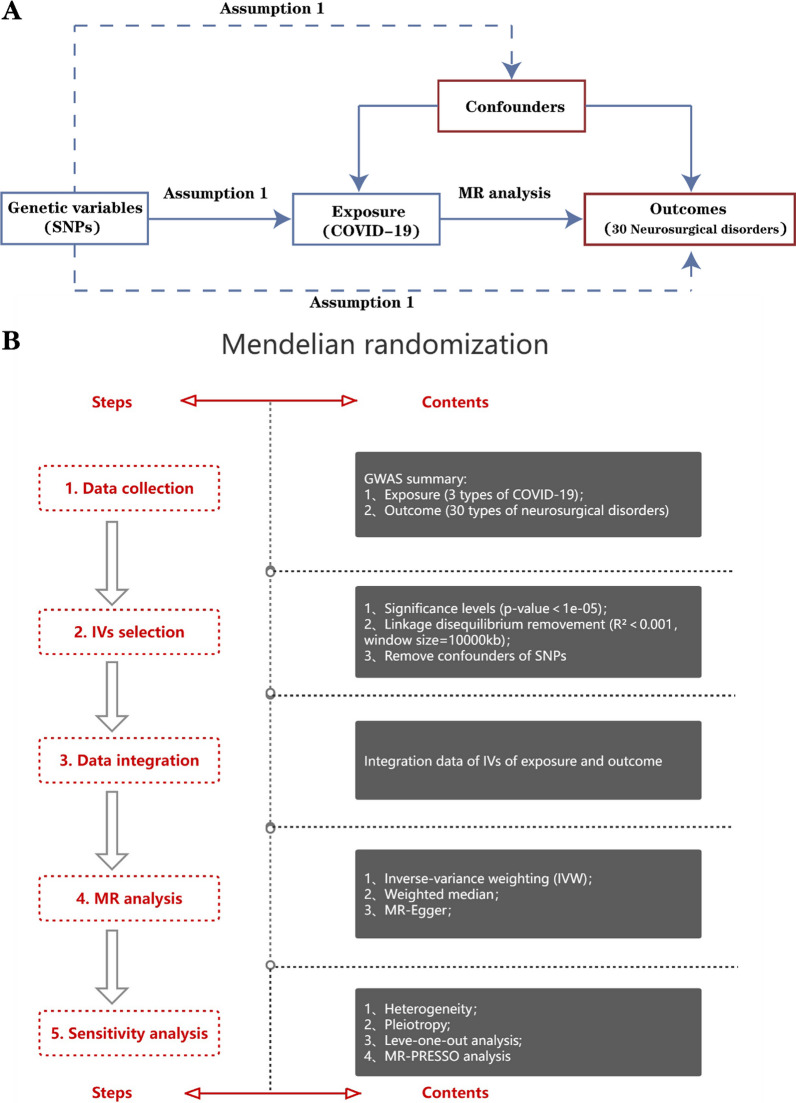
Study design. **A** Three key assumptions of the MR analysis. **B** The flowchart of the MR study. COVID‐19, coronavirus disease‐2019; MR, Mendelian randomization; GWAS, genome‐wide association study

Figure 1 reveals the overall schematic diagram of the study, where Fig. 1A reveals the three basic assumptions and Fig. 1B describes the design process of the study.

### The genome‐wide association study (GWAS) summary data sets

This study draws on data from the publicly available GWAS database (https://gwas.mrcieu.ac.uk/) [17], which classifies COVID-19 into three types, including critical COVID-19, hospitalized COVID-19, and SARS-CoV-2 infection. The pooled results <>for 30 common neurosurgical disorders in five categories were drawn from the GWAS dataset, all participants were European, and all raw data had been ethically approved. The term “Critical COVID-19” pertains to individuals with severe COVID-19 symptoms necessitating respiratory assistance or resulting in mortality, while population controls are individuals without the disease. “Hospitalized COVID-19” serves as a measure of disease severity and serves as a reference for patients admitted to hospitals with COVID-19, with population controls being individuals without the disease. “SARS-CoV-2 infection” provides an overview of the general susceptibility of the population to COVID-19, with population controls being individuals without the disease. Table 1 provides a summary of the GWAS databases encompassing 30 distinct neurosurgical diseases.

**Table 1:** Summary of the neurosurgical disorders datasets

Neurosurgical disorders	Diseases	Cases	Controls	Sample size	SNPs	Population	Year	Dataset
Cerebrovascular diseases	Trigeminal neuralgia	800	195,047	195,847	16,380,408	European	2021	finn-b-G6_TRINEU
	Epilepsy	4,382	453,928	458,310	24,186,492	European	2021	ebi-a-GCST90018840
	Parkinson’s disease	2,638	477,380	480,018	24,194,622	European	2021	ebi-a-GCST90018894
	Alzheimer’s disease	39,106	46,828	487,511	20,921,626	European	2022	ebi-a-GCST90027158
	Major depressive disorder	7,264	49,373	56,637	11,498,420	European	2021	ebi-a-GCST90086059
	Obsessive Compulsive Disorder	26,888	7,037	33,925	8,409,517	European	2017	ieu-a-1189
Functional diseases	Stroke	40,585	406,111	446,696	7,633,440	European	2018	ebi-a-GCST005838
	Intracerebral hemorrhage	1,935	471,578	473,513	24,191,284	European	2021	ebi-a-GCST90018870
	Subarachnoid hemorrhage	1,693	471,562	473,255	24,191,735	European	2021	ebi-a-GCST90018923
	Transient ischemic attack	8,835	205,799	214,634	16,380,437	European	2021	finn-b-G6_TIA
	Cerebral infarction	2,353	358,841	361,194	10,889,323	European	2018	ukb-d-I63
	Cerebral aneurysm	945	472,738	473,683	24,191,145	European	2021	ebi-a-GCST90018815
Spinal and spinal cord disease	Cervical spondylosis	3,352	481,246	484,598	9,587,836	NA	2021	ebi-a-GCST90038693
	Spinal canal stenosis	9,660	445,127	454,787	24,182,979	European	2021	ebi-a-GCST90018922
	spinal meningioma	118	218,674	218,792	16,380,466	European	2021	finn-b-CD2_BENIGN_MENINGES_SPINAL
	Spinal osteochondrosis	183	164,682	164,865	16,380,216	European	2021	finn-b-M13_SPINALOSTEOCHON
	Intracranial and intraspinal abscess	141	217,485	217,626	16,380,461	European	2021	finn-b-G6_CNSABSC
	Cervical spinal cord and nerve injuries	254	215,476	215,730	16,380,463	European	2021	finn-b-ST19_INJURY_NERVES_SPINAL_CORD_NECK_LEVEL
Central nervous system neoplasms	Glioblastoma	91	218,701	218,792	16,380,466	European	2021	finn-b-C3_GBM
	Benign meningioma	1,147	217,645	218,792	16,380,466	European	2021	finn-b-CD2_BENIGN_MENINGES_CEREBRAL
	Malignant meningioma	640	218,152	218,792	16,380,466	European	2021	finn-b-C3_MENINGES
	Pituitary adenoma and craniopharyngioma	735	218,057	218,792	16,380,466	European	2021	finn-b-CD2_BENIGN_PITUITARY_CRANIPHAR
	Benign neoplasm of brain and other parts of CNS	923	217,869	218,792	16,380,466	European	2021	finn-b-CD2_BENIGN_BRAIN_CNS
	Malignant neoplasm of brain and other parts of CNS	198	218,594	218,792	16,380,466	European	2021	finn-b-C3_SPINAL_CORD_CRANIAL_AND_OTHER_CNS
Other brain diseases	Hydrocephalus	749	205,799	206,548	16,380,404	European	2021	finn-b-G6_HYDROCEPH
	Craniosynostosis	405	218,387	218,792	16,380,466	European	2021	finn-b-Q17_CRANIOSYNOSTOSIS
	Concussion	10,527	136,576	147,103	16,380,074	European	2021	finn-b-ST19_CONCUSSION
	Diffuse brain injury	656	136,576	137,232	16,379,965	European	2021	finn-b-ST19_DIFFU_BRAIN_INJURY
	Focal brain injury	1,065	136,576	137,641	16,379,970	European	2021	finn-b-ST19_FOCAL_BRAIN_INJURY
	Congenital malformations of the nervous system	258	218,534	218,792	16,380,466	European	2021	finn-b-Q17_CONGEN_MALFO_NERVOUS_SYSTEM

### Selection of IVs

To confirm that there were enough IVs for COVID-19 to keep statistical power, we chose SNPs that were strongly associated with COVID-19 as IVs (*p* < 1e−5). Subsequently, we used *r*^2^ < 0.001 and Kb > 10,000 as thresholds to remove chain disequilibrium reactions (LD), thus guaranteeing the independence of IVs. To address the second hypothesis in the MR analysis, we use the PhenoScannerV2 database (http://www.Phenoscanner.Medschl.CAM.Ac.UK/) [18] to remove potential confounders, for instance, body mass index [19], diastolic blood pressure, systolic blood pressure [20], hypertension [21], coronary artery disease [22], treatment with warfarin [23], treatment with simvastatin [24], high cholesterol, and LDL cholesterol [25]. To further harmonize the effect alleles of exposure and outcome data sets, the selection of IVs should also exclude palindromic SNPs. To meet the first hypothesis of the MR analysis, we use R^2^ as a genetic tool to clarify the proportion of the variance of the trait, and the R^2^ statistic uses the formula (R^2^ = 2 × (1-maf) × maf × (β/SD)^2^). Calculate the F statistic to evaluate the robustness of individual SNPs. When the F statistic exceeds 10, SNPs are considered to be unaffected by weak instrumental variable bias [26]. The F statistic uses the formula F = [R^2^(N − 1 − K)] /[(1 − R^2^) × K]). K: the quantity of variants, N: the size of the sample size.

### MR analysis

We adopted three MR methods, including inverse variance weighting (IVW), weighted median, and Mendelian randomization-Egger (MR-Egger), to examine the causal association between COVID-19 and neurosurgical diseases. The IVW model was primarily utilized to assess the causal relationship between COVID-19 and neurosurgical disease. The IVW disregards the presence of an intercept term in regression and employs the reciprocal of outcome variance as the weight for fitting. It evaluates causal effects by amalgamating ratio estimates for each SNP [27]. The weighted median model assigns greater importance to accurate instrumental variables (IVs) and is capable of producing unbiased estimates even when up to 50% of the information is come from invalid IVs. MR-Egger, on the other hand, enables the estimation of causal effects by examining the slope coefficient of MR-Egger regression, while the intercept of MR-Egger regression can be employed to access the average level of pleiotropy [28, 29]. However, it should be noted that both the weighted median and MR-Egger models exhibit lower statistical power compared to the IVW model. Consequently, the IVW model is the most commonly employed method for obtaining variance-specific causal estimates in two-sample Mendelian randomization analyses.

### Sensitivity analysis

We used a variety of sensitivity analysis methods to evaluate the robustness of the causal relationship between COVID-19 and neurosurgical disease. First, we use Cochran’Q statistics to evaluate heterogeneity. When the *p*-value of the heterogeneity test result is under 0.05, it indicates the existence of heterogeneity, and we need to further test the random effects model as the main method [30]. We then used the MR-Egger intercept for a pleiotropy test, after which a leave-one-out analysis was used to evaluate whether SNPs produced significant results, thereby removing the promiscuous SNPs one by one [12].

### Statistically

The statistical analyses were performed utilizing the "TwoSampleMR" software package in R version 4.2.0. A robust association was determined if the result maintained significant after applying the Bonferroni correction (*p* < 0.05). Additionally, associations were considered robust if at least two different MR analyses yielded significant results (*p* < 0.05). Although the *p*-value exceeded the significance threshold for correction, evidence suggestive of an association was still considered if *p* < 0.05 in at least one method. In terms of sensitivity analysis, significant heterogeneity and horizontal pleiotropy were indicated when *p* < 0.05.

## Results

### Genetic IVs for COVID‐19

According to the criteria we established, 26, 32, and 45 SNPs were identified as IVs to analyze associations between neurological diseases and COVID-19, including critically ill COVID‐19, hospitalized COVID‐19, and SARS‐CoV‐2 infection, respectively. Additional file 1: Table S1 provides comprehensive details about the SNPs screening process. Next, we will introduce the analysis results of the causal association between COVID-19 and neurosurgical disorders specifically.

### Causal effects of critically ill COVID‐19 on neurosurgical diseases

**Fig. 2:**
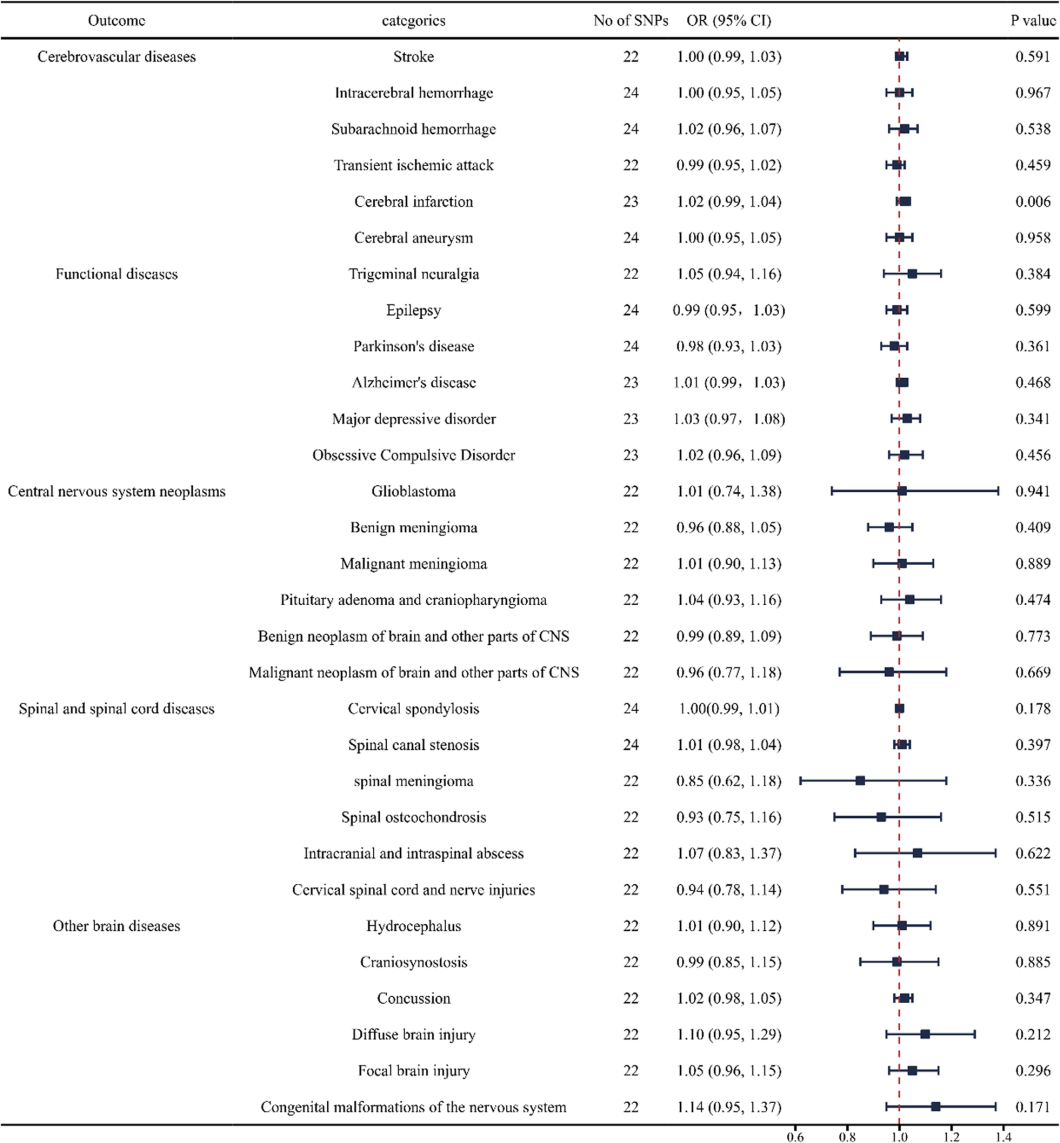
Forest plot of inverse‐variance weighted MR analyses of critically ill COVID‐19 on the risk of neurosurgical disorders. CI, confidence interval; SNP, single nucleotide polymorphism; COVID‐19, coronavirus disease‐2019; MR, Mendelian randomization; IVW, inverse‐variance weighted; OR, odds ratio

We discovered a robust association between the genetic susceptibility of critically ill COVID-19 and an increased risk of cerebral infarction in cerebrovascular diseases (odds ratio [OR] = 1.02; 95% confidence interval [CI] 0.99, 1.04). And we found a weak association between genetic susceptibility of critically ill COVID-19 and an increased risk of diffuse brain injury in other brain diseases (OR = 1.10; CI 0.95, 1.29). However, no causal relationship has been found between critically ill COVID-19 and other neurosurgical diseases (Fig. 2 and Additional file 2: Table S2).

### Causal effects of hospitalized COVID‐19 on neurosurgical diseases

**Fig. 3:**
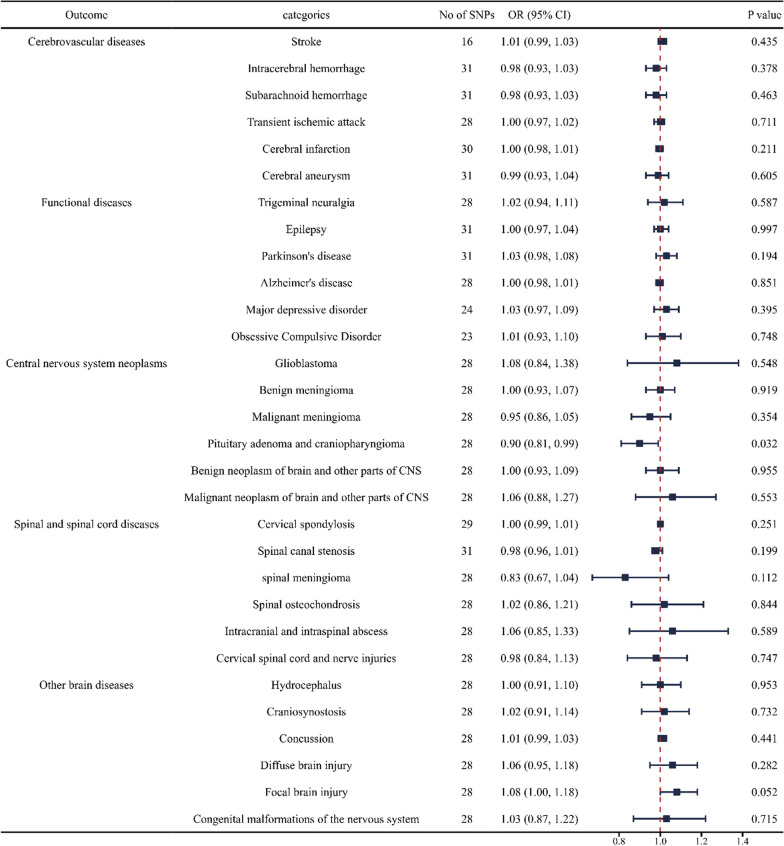
Forest plot of inverse‐variance weighted MR analyses of hospitalized COVID‐19 on the risk of neurosurgical disorders. CI, confidence interval; SNP, single nucleotide polymorphism; COVID‐19, coronavirus disease‐2019; MR, Mendelian randomization; IVW, inverse‐variance weighted; OR, odds ratio

We discovered a robust association between the genetic susceptibility of hospitalized COVID-19 and a decreased risk of pituitary adenoma and craniopharyngioma in central nervous system neoplasms (OR = 0.90; CI 0.81, 0.99). And we found a weak association between genetic susceptibility of hospitalized COVID-19 and an increased risk of focal brain injury in other brain diseases (OR = 1.08; CI 1.00, 1.18). However, no causal relationship has been found between hospitalized COVID-19 and other neurosurgical diseases (Fig. 3 and Additional file 3: Table S3).

### Causal effects of SARS‐CoV‐2 infection on neurosurgical diseases

**Fig. 4:**
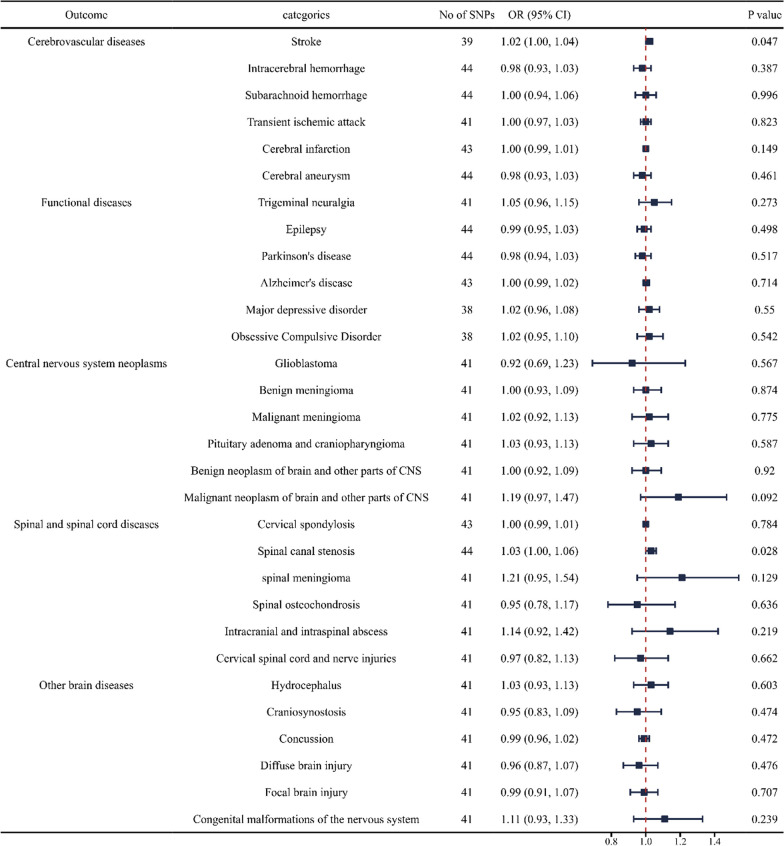
Forest plot of inverse‐variance weighted MR analyses of SARS‐CoV‐2 infection on the risk of neurosurgical disorders. CI, confidence interval; SNP, single nucleotide polymorphism; COVID‐19, coronavirus disease‐2019; MR, Mendelian randomization; IVW, inverse‐variance weighted; OR, odds ratio

We discovered a robust association between the genetic susceptibility of SARS‐CoV‐2 infection and an increased risk of stroke in cerebrovascular diseases (OR = 1.02; CI 1.00, 1.04). And we found a weak association between genetic liabilities of SARS‐CoV‐2 infection and an increased risk of spinal canal stenosis in spinal and spinal cord diseases (OR = 1.03; CI 1.00, 1.06). Meanwhile, the genetic susceptibility to SARS‐CoV‐2 infection had weak causal associations with the decreased risk for epilepsy in functional diseases (OR = 0.99; CI 0.95, 1.03). However, no causal relationship has been found between SARS‐CoV‐2 infection and other neurosurgical diseases (Fig. 4 and Additional file 4: Table S4).

### Sensitivity analysis

The sensitivity analysis consequences were utilized to validate the robustness of the causal associations between COVID-19 and neurosurgical diseases. For heterogeneity test, *p*-value > 0.05 indicates that there is no heterogeneity in MR analysis. The heterogeneity test in this study demonstrated no heterogeneity in the majority of MR analyses (Cochran’s Q statistic, *p*‐value > 0.05). To ensure the reliability of the MR analysis consequences, a random effects model was employed for groups with a *p*-value ≤ 0.05. The MR-Egger intercept is employed to access the pleiotropy test. When *p* < 0.05, it indicates the existence of pleiotropy, that is, the MR analysis results are unstable. Throughout our analysis results, only in the MR analysis of SARS‐CoV‐2 infection and epilepsy, the *p*-value of pleiotropy test was lower than 0.05, that is, the analysis result was unstable, and the *p*-value of all other results was above 0.05, indicating that the MR analysis results were robust (Additional file 2: Table S2, Additional file 3: Table S3, Additional file 4: Table S4).

## Discussion

COVID-19 has caused many physical illnesses for people around the world, and many studies have identified it and neurological disorders as risk factors for each other; however, the mechanism of its interaction is unclear, and systematic research and analysis results are lacking [29]. Therefore, we used MR analysis to systematically and comprehensively study the causal relationship between COVID-19 and neurosurgical disorders, so as to guide the prevention, treatment and later rehabilitation of patients with related diseases.

Our study found causal relationships between COVID-19 and the risk of multiple diseases in neurosurgery. The genetic predisposition of individuals with critically ill COVID-19 is linked to a heightened likelihood of developing cerebral infarction within the context of cerebrovascular disease; the genetic susceptibility of hospitalized COVID-19 patients is associated with a diminished risk of pituitary tumors and craniopharyngiomas within the realm of nervous system tumors. Additionally, the genetic susceptibility to SARS-CoV-2 infection is correlated with an elevated risk of stroke within the context of cerebrovascular disease. In addition, some neurosurgical diseases are weakly associated with COVID-19, including spinal stenosis, epilepsy, diffuse brain injury, and focal brain injury. In general, diseases in various neurosurgical subspecialties may be associated with COVID-19, especially cerebrovascular diseases and brain tumors.

Although COVID-19 mainly affects the lungs, many studies have reported various direct or indirect associations between COVID-19 and the occurrence of cerebral infarction. COVID-19 can lead to coagulation dysfunction and inflammation in various patients, promote the disorder of blood clotting function, and thus cause venous thromboembolism and lead to brain infarction [31, 32]. Some studies have found that cerebral artery occlusion and infarction occurred in infants with critically ill COVID-19 due to D-dimer elevation and coagulation dysfunction [33]. It is also believed that D-dimer level is helpful in evaluating asymptomatic COVID-19 with acute myocardial infarction [34]. When COVID-19 patients suffer from cerebral infarction meanwhile, it is essential to develop an individualized treatment plan for them. In addition to the conventional antibacterial treatment and the treatment of eliminating phlegm and relieving asthma for COVID-19 patients, anti-coagulation treatment should be actively applied, blood pressure and blood lipid should be measured, and the immunity of patients should be enhanced [8]. In conclusion, genetic susceptibility of critically ill COVID-19 infection is strongly linked to the increased risk of cerebral infarction, especially in the elderly and infants, and prevention and treatment of related patients is necessary.

SARS-CoV-2 is a virus that can cause cell damage and death. Its structural protein is treated by the transmembrane protease serine 2 (TMPRSS2), which promotes the virus to bind to angiotensin-converting enzyme 2 (ACE2) and enter the epithelial alveolar cells of the host [35]. Epithelial cells then release replicating viruses, upregulate IL-1β of macrophages and trigger the activation of inflammasome, which causes local pulses of tumor necrosis factor (TNF), IL-1β, IL-8, and MCP-1, promoting the continuous increase of IL-6 and perpetuating the inflammatory process [36]. SARS-CoV-2 patients have an excessive inflammatory response, and increased levels of D-dimer, C-reactive protein, and IL-6 are often seen in patients [37]. In addition, SARS-CoV-2 can bind to ACE2-expressing cells to promote local inflammation, resulting in microcirculatory dysfunction [38]. In this state of hyperinflammation, locally activated platelets can induce the release of neutrophil extrinsic traps, thereby activating the exogenous clotting process of thrombin formation [39]. Some researchers have also found that complement factor C5a increases in proportion to the severity of COVID-19, and C5aR1 receptor expression is also increased in the blood, suggesting that complement activation may also be a cause of persistent inflammation and clotting in COVID-19 patients [40]. Since the COVID-19 pandemic, studies on the correlation between COVID-19 and ischemic stroke have never been interrupted. The above mechanisms all help us explain the pathogenesis of ischemic stroke in COVID-19 patients, and this study further provides genetic evidences of the correlation between them.

Another significant result is that the genetic predisposition to hospitalized COVID-19 may reduce the risk of pituitary tumors and craniopharyngiomas. More and more researchers are focusing on the possible mechanisms of tumor regression, including in patients with COVID-19 [41]. One study found that a pituitary tumor patient with COVID-19 was hospitalized for antiviral therapy and steroid therapy for three months, and brain MRI showed that the pituitary tumor low signal disappeared, visual impairment and headache symptoms were greatly reduced [42]. There was also a patient with a pituitary tumor whose surgery was postponed for half a year due to COVID-19, but unexpectedly found that most of the pituitary tumor had subsided [43]. The degeneration of pituitary adenomas described above is uncommon, so we hypothesize that the patient is due to anti-tumor immune response caused by SARS-CoV-2 infection, prompting pathogen-specific T cells to cross-react with tumor antigens, and activating natural killer cells through inflammatory cytokines produced by viral infection [44]. Since there have been no direct reports of the association between COVID-19 and craniopharyngioma, our study may provide reference for the treatment of patients with craniopharyngioma hospitalized with COVID-19 in the future.

In addition, this study found a weak association between COVID-19 and epilepsy, brain injury, and spinal canal stenosis, and a review of researchers’ reports on related studies showed mixed results. Some studies suggest that the incidence of acute seizures caused by COVID-19 is less than 1%, so it is not enough to prove the correlation with epilepsy [45, 46]. Researchers also included 5,700 patients with epilepsy, and only 14 patients were diagnosed with COVID-19 [47], so patients with epilepsy did not have a significantly increased risk of contracting COVID-19. As for brain injury, by monitoring the serum markers of brain injury (NfL) and neuro-collagen fibrillary acidic protein (GFAP) in patients, researchers have found that brain injury is a common consequence of both COVID-19 and common influenza, and therefore lacks specificity [48]. No causal association between COVID-19 and spinal stenosis has been reported. All in all, these neurosurgical diseases with weak associations with COVID-19 need more clinical studies and molecular mechanisms to explore and verify.

Of course, there are some limitations to our study, such as the possibility of racial differences because our results are mainly based on European populations; and our analysis of the causal relationship between COVID-19 and neurosurgical disease may not be able to completely exclude potential confounders.

In summary, this study is the first to systematically report causal relationships between the genetic susceptibility of COVID-19 and 30 neurosurgical disorders using MR analysis. In strict accordance with the requirements of MR analysis, we screened the instrumental variables, removed the linkage imbalance and exclusive assumptions. Finally, robust causal associations were revealed between critically ill COVID-19 and cerebral infarction, hospitalized COVID-19 and pituitary tumor and craniopharyngioma, and SARS-CoV-2 infection and stroke. Our study has vital guiding significance for the health supervision of the nervous system of COVID-19 patients, especially for the risk assessment and timely treatment of cerebrovascular diseases and brain tumors. In the future, we will also establish a systematic screening system, and carry out relevant basic research to explore specific molecular mechanisms, and strive to acquire early detection and early treatment, so as to enhance the survival rate and quality of life of patients.

## Conclusions

This research revealed that the genetic susceptibility to critically ill COVID-19 infection may increase the risk of cerebral infarction, and the genetic susceptibility to SARS-CoV-2 infection may increase the risk of stroke. Conversely, the genetic susceptibility to hospitalized COVID-19 infection may decrease the risk of pituitary tumors and craniopharyngiomas. Furthermore, there exists suggestive evidence indicating a weak correlation between the aforementioned neurosurgical diseases and COVID-19. It is plausible that a genetic susceptibility toward critically ill COVID-19 infection could heighten the likelihood of experiencing diffuse brain injury, while a genetic susceptibility toward hospitalized COVID-19 infection may elevate the risk of focal brain injury. Additionally, individuals with a genetic susceptibility to SARS-CoV-2 infection may face an increased risk of spinal stenosis, but may decrease the risk of epilepsy.

### Supplementary Information

**Additional file 1: Table S1.** Single-nucleotide polymorphisms associated with COVID-19 (*P* < 1 × 10^−5^). √ is confounding factor, × is non-confounding factor.

**Additional file 2: Table S2.** Associations between genetically predicted critically ill COVID-19 and 30 neurosurgical disorders in sensitivity analyses using the weighted-median and MR-Egger methods.

**Additional file 3: Table S3.** Associations between genetically predicted hospitalized COVID-19 and 30 neurosurgical disorders in sensitivity analyses using the weighted-median and MR-Egger methods.

**Additional file 4: Table S4.** Associations between genetically predicted SARS‐CoV‐2 infection and 30 neurosurgical disorders in sensitivity analyses using the weighted-median and MR-Egger methods.

## Data Availability

All data are available on public repositories, which are listed in the main context. All data included in this study are available by contacting the corresponding authors.
