# Supplementary material for: Causal associations of COVID‐19 on neurosurgical diseases risk: a Mendelian randomization study
Source: Hum Genomics. 2024 Feb 5;18:13. doi: 10.1186/s40246-024-00575-y (PMC10840232; doi:10.1186/s40246-024-00575-y)
Supplement: Supplementary file 1 — Additional file 1: Table S1. Single-nucleotide polymorphisms associated with COVID-19 (P < 1 × 10^−5^). √ is confounding factor, × is non-confounding factor. [file 40246_2024_575_MOESM1_ESM.docx]

| Exposure | SNP | Effect_allele | Other_allele | Beta | Se | P-value | MAF | Confounding factors |
| --- | --- | --- | --- | --- | --- | --- | --- | --- |
| *critically ill COVID‐19* | rs4076440 | G | A | 0.301866 | 0.0682837 | 9.83E-06 | 0.11 | *×* |
| *critically ill COVID‐19* | rs302808 | T | C | 0.257846 | 0.0564942 | 5.02E-06 | 0.17 | *×* |
| *critically ill COVID‐19* | rs12622794 | A | G | -0.193706 | 0.0429963 | 6.63E-06 | 0.27 | *×* |
| *critically ill COVID‐19* | rs73064425 | T | C | 0.762813 | 0.0669807 | 4.77E-30 | 0.08 | *×* |
| *critically ill COVID‐19* | rs11742461 | T | C | -0.193402 | 0.0427783 | 6.16E-06 | 0.44 | *×* |
| *critically ill COVID‐19* | rs299284 | T | C | 0.29199 | 0.0632056 | 3.84E-06 | 0.17 | *×* |
| *critically ill COVID‐19* | rs143334143 | A | G | 0.615083 | 0.0716179 | 8.82E-18 | 0.08 | *×* |
| *critically ill COVID‐19* | rs854080 | A | G | -0.208241 | 0.0471267 | 9.93E-06 | 0.29 | *×* |
| *critically ill COVID‐19* | rs12705891 | C | T | 0.275394 | 0.0434621 | 2.35E-10 | 0.36 | *×* |
| *critically ill COVID‐19* | rs10087754 | A | T | -0.193533 | 0.0432237 | 7.55E-06 | 0.41 | *×* |
| *critically ill COVID‐19* | rs116834121 | A | G | -0.692665 | 0.154557 | 7.41E-06 | 0.01 | *×* |
| *critically ill COVID‐19* | rs7852250 | C | T | -0.394795 | 0.0867419 | 5.33E-06 | 0.19 | *×* |
| *critically ill COVID‐19* | rs4366152 | C | T | 0.210935 | 0.0464797 | 5.67E-06 | 0.27 | *×* |
| *critically ill COVID‐19* | rs111832785 | A | G | -0.585979 | 0.130327 | 6.92E-06 | ＜0.01 | *×* |
| *critically ill COVID‐19* | rs78594643 | T | C | -0.723176 | 0.160873 | 6.95E-06 | 0.01 | *×* |
| *critically ill COVID‐19* | rs138476485 | T | C | 0.774948 | 0.175243 | 9.77E-06 | ＜0.01 | *×* |
| *critically ill COVID‐19* | rs11223253 | G | A | 0.465613 | 0.104161 | 7.82E-06 | 0.10 | *×* |
| *critically ill COVID‐19* | rs10735079 | A | G | 0.25784 | 0.0456738 | 1.65E-08 | 0.26 | *×* |
| *critically ill COVID‐19* | rs817724 | T | C | -0.26064 | 0.0576721 | 6.20E-06 | 0.19 | *×* |
| *critically ill COVID‐19* | rs4646632 | T | C | -0.466106 | 0.0924663 | 4.64E-07 | 0.08 | *×* |
| *critically ill COVID‐19* | rs4965778 | G | A | -0.197466 | 0.0443926 | 8.66E-06 | 0.45 | *×* |
| *critically ill COVID‐19* | rs114277682 | G | T | -0.556336 | 0.122332 | 5.42E-06 | 0.03 | *×* |
| *critically ill COVID‐19* | rs2109069 | A | G | 0.305623 | 0.0440513 | 3.98E-12 | 0.21 | *×* |
| *critically ill COVID‐19* | rs2080997 | G | C | -0.196722 | 0.0432441 | 5.39E-06 | 0.28 | *×* |
| *critically ill COVID‐19* | rs1233039 | T | C | 0.30103 | 0.0665053 | 6.00E-06 | 0.10 | *×* |
| *critically ill COVID‐19* | rs2236757 | G | A | -0.251062 | 0.0460548 | 5.00E-08 | 0.37 | *×* |
|  |  |  |  |  |  |  |  |  |
| *hospitalized COVID‐19* | rs112317747 | C | T | 0.58545 | 0.13119 | 8.10E-06 | 0.03348 | *×* |
| *hospitalized COVID‐19* | rs56229346 | A | C | 0.94905 | 0.20583 | 4.01E-06 | 0.115 | *×* |
| *hospitalized COVID‐19* | rs2224986 | T | C | -0.31065 | 0.069603 | 8.07E-06 | 0.1809 | *×* |
| *hospitalized COVID‐19* | rs2034831 | C | A | 0.37803 | 0.081371 | 3.39E-06 | 0.1543 | *×* |
| *hospitalized COVID‐19* | rs77695931 | A | G | 0.61946 | 0.1394 | 8.84E-06 | 0.1248 | *×* |
| *hospitalized COVID‐19* | rs112862616 | A | C | 0.87515 | 0.19319 | 5.90E-06 | 0.1775 | *×* |
| *hospitalized COVID‐19* | rs13062942 | A | G | 0.25854 | 0.056152 | 4.14E-06 | 0.5946 | *×* |
| *hospitalized COVID‐19* | rs13079478 | T | G | 0.34901 | 0.062872 | 2.84E-08 | 0.1857 | *×* |
| *hospitalized COVID‐19* | rs1705826 | C | G | 0.26077 | 0.056157 | 3.42E-06 | 0.5516 | *×* |
| *hospitalized COVID‐19* | rs79835427 | G | T | 0.89988 | 0.20166 | 8.11E-06 | 0.1669 | *×* |
| *hospitalized COVID‐19* | rs76488148 | T | G | 0.37445 | 0.083226 | 6.82E-06 | 0.1631 | *×* |
| *hospitalized COVID‐19* | rs114776680 | A | G | 0.688 | 0.15464 | 8.63E-06 | 0.1216 | *×* |
| *hospitalized COVID‐19* | rs142501681 | A | G | 1.3526 | 0.26785 | 4.42E-07 | 0.01456 | *×* |
| *hospitalized COVID‐19* | rs4478338 | G | T | -0.32525 | 0.069635 | 3.00E-06 | 0.1921 | *×* |
| *hospitalized COVID‐19* | rs112479169 | T | C | 0.66698 | 0.14044 | 2.04E-06 | 0.02502 | *√* |
| *hospitalized COVID‐19* | rs79385393 | A | C | 1.1016 | 0.21998 | 5.50E-07 | 0.1207 | *×* |
| *hospitalized COVID‐19* | rs6967210 | C | T | 0.4224 | 0.093758 | 6.63E-06 | 0.06281 | *×* |
| *hospitalized COVID‐19* | rs138608529 | T | A | 1.4358 | 0.3244 | 9.60E-06 | 0.009402 | *×* |
| *hospitalized COVID‐19* | rs332040 | A | G | 0.19471 | 0.041236 | 2.34E-06 | 0.5099 | *√* |
| *hospitalized COVID‐19* | rs183209580 | C | T | 1.1073 | 0.24937 | 8.97E-06 | 0.2035 | *×* |
| *hospitalized COVID‐19* | rs139368606 | G | T | 0.92511 | 0.20681 | 7.70E-06 | 0.1149 | *×* |
| *hospitalized COVID‐19* | rs71480372 | A | T | 0.26497 | 0.056125 | 2.35E-06 | 0.6479 | *×* |
| *hospitalized COVID‐19* | rs5016035 | G | T | 0.36247 | 0.079125 | 4.63E-06 | 0.4617 | *×* |
| *hospitalized COVID‐19* | rs75256341 | A | C | 0.83277 | 0.1559 | 9.20E-08 | 0.1224 | *×* |
| *hospitalized COVID‐19* | rs7397549 | C | T | 0.26527 | 0.059259 | 7.59E-06 | 0.3975 | *×* |
| *hospitalized COVID‐19* | rs2649134 | C | T | -0.79498 | 0.176 | 6.27E-06 | 0.8136 | *×* |
| *hospitalized COVID‐19* | rs563015997 | T | C | 0.9757 | 0.20425 | 1.78E-06 | 0.1279 | *×* |
| *hospitalized COVID‐19* | rs77055952 | G | A | 0.4404 | 0.096982 | 5.60E-06 | 0.1364 | *×* |
| *hospitalized COVID‐19* | rs72779789 | C | G | 0.46698 | 0.10395 | 7.05E-06 | 0.1467 | *×* |
| *hospitalized COVID‐19* | rs141909101 | T | C | 0.89109 | 0.17816 | 5.68E-07 | 0.1263 | *×* |
| *hospitalized COVID‐19* | rs9890316 | A | G | -0.27018 | 0.060496 | 7.96E-06 | 0.4048 | *×* |
| *hospitalized COVID‐19* | rs142257532 | C | T | 0.55954 | 0.12316 | 5.54E-06 | 0.1301 | *×* |
| *hospitalized COVID‐19* | rs76253189 | G | C | 0.49549 | 0.11099 | 8.04E-06 | 0.1367 | *×* |
| *hospitalized COVID‐19* | rs75994231 | T | C | 0.81065 | 0.18318 | 9.63E-06 | 0.1328 | *×* |
|  |  |  |  |  |  |  |  |  |
| *very severe respiratory confirmed COVID‐19* | rs9287218 | C | A | -0.28672 | 0.062974 | 5.29E-06 | 0.04678 | *×* |
| *very severe respiratory confirmed COVID‐19* | rs4076440 | G | A | 0.20322 | 0.045231 | 7.02E-06 | 0.09872 | *×* |
| *very severe respiratory confirmed COVID‐19* | rs113488799 | C | T | 0.21399 | 0.041404 | 2.36E-07 | 0.3174 | *×* |
| *very severe respiratory confirmed COVID‐19* | rs28815269 | A | G | -0.19391 | 0.041747 | 3.40E-06 | 0.6337 | *×* |
| *very severe respiratory confirmed COVID‐19* | rs1453205 | T | C | 0.16872 | 0.037106 | 5.44E-06 | 0.5234 | *×* |
| *very severe respiratory confirmed COVID‐19* | rs114380132 | A | G | 0.65074 | 0.14025 | 3.49E-06 | 0.01066 | *×* |
| *very severe respiratory confirmed COVID‐19* | rs1064213 | A | G | 0.13498 | 0.027803 | 1.20E-06 | 0.4482 | *√* |
| *very severe respiratory confirmed COVID‐19* | rs115207228 | T | C | 0.77002 | 0.14248 | 6.51E-08 | 0.02505 | *×* |
| *very severe respiratory confirmed COVID‐19* | rs12330486 | A | G | -0.24105 | 0.049906 | 1.36E-06 | 0.09819 | *×* |
| *very severe respiratory confirmed COVID‐19* | rs35081325 | T | A | 0.68803 | 0.046813 | 6.70E-49 | 0.07389 | *×* |
| *very severe respiratory confirmed COVID‐19* | rs147160803 | A | G | 0.90799 | 0.18586 | 1.03E-06 | 0.01234 | *×* |
| *very severe respiratory confirmed COVID‐19* | rs114969787 | T | C | 0.3053 | 0.068589 | 8.54E-06 | 0.03751 | *×* |
| *very severe respiratory confirmed COVID‐19* | rs146410305 | T | G | 0.58565 | 0.12315 | 1.98E-06 | 0.01861 | *×* |
| *very severe respiratory confirmed COVID‐19* | rs79833209 | T | C | 0.47383 | 0.09498 | 6.08E-07 | 0.02528 | *×* |
| *very severe respiratory confirmed COVID‐19* | rs143334143 | A | G | 0.33972 | 0.045476 | 8.01E-14 | 0.09934 | *×* |
| *very severe respiratory confirmed COVID‐19* | rs4715051 | G | A | -0.12543 | 0.027713 | 6.01E-06 | 0.5021 | *×* |
| *very severe respiratory confirmed COVID‐19* | rs2237698 | T | C | 0.23871 | 0.042315 | 1.69E-08 | 0.08429 | *×* |
| *very severe respiratory confirmed COVID‐19* | rs7791022 | G | A | 0.50766 | 0.10808 | 2.64E-06 | 0.01819 | *×* |
| *very severe respiratory confirmed COVID‐19* | rs622568 | C | A | 0.22722 | 0.037705 | 1.68E-09 | 0.1391 | *×* |
| *very severe respiratory confirmed COVID‐19* | rs10216545 | A | G | 0.16791 | 0.037452 | 7.35E-06 | 0.46 | *×* |
| *very severe respiratory confirmed COVID‐19* | rs10087789 | G | C | -0.12781 | 0.028364 | 6.60E-06 | 0.5731 | *×* |
| *very severe respiratory confirmed COVID‐19* | rs2781267 | A | G | -0.13682 | 0.029736 | 4.20E-06 | 0.3123 | *×* |
| *very severe respiratory confirmed COVID‐19* | rs1566837 | A | G | -0.17643 | 0.036856 | 1.69E-06 | 0.4588 | *×* |
| *very severe respiratory confirmed COVID‐19* | rs550057 | T | C | 0.18539 | 0.040739 | 5.35E-06 | 0.2751 | *√* |
| *very severe respiratory confirmed COVID‐19* | rs787642 | A | C | -0.14372 | 0.030484 | 2.42E-06 | 0.7474 | *×* |
| *very severe respiratory confirmed COVID‐19* | rs118052809 | A | T | 0.45735 | 0.10249 | 8.10E-06 | 0.01624 | *×* |
| *very severe respiratory confirmed COVID‐19* | rs12806161 | A | G | -0.1292 | 0.02853 | 5.94E-06 | 0.4304 | *×* |
| *very severe respiratory confirmed COVID‐19* | rs11034760 | G | A | 0.21805 | 0.048783 | 7.83E-06 | 0.06164 | *×* |
| *very severe respiratory confirmed COVID‐19* | rs2597569 | C | T | -0.16404 | 0.035361 | 3.50E-06 | 0.532 | *×* |
| *very severe respiratory confirmed COVID‐19* | rs2298703 | A | G | 0.21119 | 0.047735 | 9.68E-06 | 0.1911 | *×* |
| *very severe respiratory confirmed COVID‐19* | rs2269899 | T | C | 0.18841 | 0.029553 | 1.83E-10 | 0.6777 | *×* |
| *very severe respiratory confirmed COVID‐19* | rs10860891 | A | C | -0.26602 | 0.041627 | 1.65E-10 | 0.8922 | *×* |
| *very severe respiratory confirmed COVID‐19* | rs10875713 | T | A | -0.20265 | 0.043396 | 3.01E-06 | 0.1817 | *×* |
| *very severe respiratory confirmed COVID‐19* | rs9533610 | G | A | -0.17468 | 0.039388 | 9.22E-06 | 0.871 | *×* |
| *very severe respiratory confirmed COVID‐19* | rs72638841 | T | G | -0.25434 | 0.056591 | 6.98E-06 | 0.08378 | *×* |
| *very severe respiratory confirmed COVID‐19* | rs77692136 | A | G | -0.37566 | 0.080814 | 3.35E-06 | 0.07918 | *×* |
| *very severe respiratory confirmed COVID‐19* | rs11634857 | A | G | -0.16323 | 0.033475 | 1.08E-06 | 0.2444 | *×* |
| *very severe respiratory confirmed COVID‐19* | rs200678047 | A | G | 0.30843 | 0.068533 | 6.78E-06 | 0.07674 | *×* |
| *very severe respiratory confirmed COVID‐19* | rs4782434 | G | A | -0.15737 | 0.034079 | 3.88E-06 | 0.7614 | *×* |
| *very severe respiratory confirmed COVID‐19* | rs9933400 | A | G | 0.57278 | 0.12303 | 3.23E-06 | 0.01847 | *×* |
| *very severe respiratory confirmed COVID‐19* | rs149399480 | T | C | 1.0539 | 0.23379 | 6.56E-06 | 0.008708 | *×* |
| *very severe respiratory confirmed COVID‐19* | rs77534576 | T | C | 0.38269 | 0.077602 | 8.17E-07 | 0.03605 | *×* |
| *very severe respiratory confirmed COVID‐19* | rs2277732 | A | C | 0.24312 | 0.02994 | 4.65E-16 | 0.3221 | *×* |
| *very severe respiratory confirmed COVID‐19* | rs10412437 | G | A | -0.39812 | 0.088909 | 7.54E-06 | 0.02145 | *√* |
| *very severe respiratory confirmed COVID‐19* | rs8111981 | G | A | -0.25048 | 0.05501 | 5.28E-06 | 0.8374 | *×* |
| *very severe respiratory confirmed COVID‐19* | rs45524632 | A | C | 0.44016 | 0.09141 | 1.47E-06 | 0.02158 | *×* |
| *very severe respiratory confirmed COVID‐19* | rs13050728 | C | T | -0.19735 | 0.029635 | 2.75E-11 | 0.6641 | *×* |
| *very severe respiratory confirmed COVID‐19* | rs5767981 | G | A | -0.169 | 0.037383 | 6.16E-06 | 0.4449 | *×* |

**Table S1** Single-nucleotide polymorphisms associated with COVID-19 (P < 1×10-5). √ is confounding factor, × is non-confounding factor.
