# Supplementary material for: Causal associations of COVID‐19 on neurosurgical diseases risk: a Mendelian randomization study
Source: Hum Genomics. 2024 Feb 5;18:13. doi: 10.1186/s40246-024-00575-y (PMC10840232; doi:10.1186/s40246-024-00575-y)
Supplement: Supplementary file 2 — Additional file 2: Table S2. Associations between genetically predicted critically ill COVID-19 and 30 neurosurgical disorders in sensitivity analyses using the weighted-median and MR-Egger methods. [file 40246_2024_575_MOESM2_ESM.docx]

| Outcome | | Weighted Median | | MR-Egger | | Pleiotropy | | Heterogeneity | |
| --- | --- | --- | --- | --- | --- | --- | --- | --- | --- |
|  |  | OR (95% CI) | P | OR (95% CI) | P | Intercept | P | Q | P |
| critically ill COVID‐19 | Trigeminal neuralgia | 1.04 (0.60, 1.82) | 0.19 | 1.02 (0.76, 1.38) | 0.88 | 0.007 | 0.87 | 20 | 0.50 |
|  | Epilepsy | 0.98 (0.93, 1.04) | 0.47 | 1.06 (0.96, 1.17) | 0.27 | -0.021 | 0.17 | 24 | 0.42 |
|  | Parkinson's disease | 0.96 (0.90, 1.03) | 0.30 | 0.99 (0.87, 1.14) | 0.91 | -0.005 | 0.80 | 16 | 0.84 |
|  | Alzheimer's disease | 1.00 (0.98，1.02) | 0.96 | 0.99 (0.95， 1.04) | 0.72 | 0.005 | 0.47 | 34 | 0.053 |
|  | Major depressive disorder | 1.02 (0.95, 1.09) | 0.65 | 1.04 (0.88, 1.24) | 0.65 | -0.004 | 0.86 | 19 | 0.66 |
|  | Obsessive Compulsive Disorder | 1.02 (0.93, 1.11) | 0.72 | 1.05 (0.90, 1.22) | 0.56 | -0.008 | 0.75 | 22 | 0.49 |
|  |  |  |  |  |  |  |  |  |  |
|  | Stroke | 1.02 (1.00, 1.04) | 0.11 | 1.02 (0.97, 1.07) | 0.51 | -0.004 | 0.61 | 35 | 0.03 |
|  | Intracerebral hemorrhage | 1.01 (0.94, 1.08) | 0.80 | 0.94 (0.81, 1.08) | 0.38 | 0.020 | 0.35 | 22 | 0.52 |
|  | Subarachnoid hemorrhage | 1.00 (0.92, 1.07) | 0.91 | 0.96 (0.83, 1.12) | 0.63 | 0.017 | 0.45 | 19 | 0.09 |
|  | Transient ischemic attack | 0.98 (0.94, 1.03) | 0.50 | 0.96 (0.87, 1.05) | 0.37 | 0.010 | 0.49 | 17 | 0.72 |
|  | Cerebral infarction | 1.00 (0.99, 1.01) | 0.04 | 1.00 (0.99, 1.01) | 0.74 | 0.000 | 0.40 | 22 | 0.44 |
|  | Cerebral aneurysm | 1.00 (0.93, 1.07) | 0.95 | 0.97 (0.83, 1.12) | 0.66 | 0.010 | 0.66 | 21 | 0.60 |
|  |  |  |  |  |  |  |  |  |  |
|  | Cervical spondylosis | 1.00(0.99, 1.01) | 0.40 | 1.00(0.99, 1.01) | 0.26 | -8.41e-05 | 0.51 | 25 | 0.36 |
|  | Spinal canal stenosis | 1.02 (0.98, 1.07) | 0.31 | 0.97 (0.90, 1.05) | 0.50 | 0.013 | 0.28 | 24 | 0.40 |
|  | spinal meningioma | 1.08 (0.72, 1.62) | 0.71 | 0.71 (0.28, 1.18) | 0.49 | 0.055 | 0.69 | 30 | 0.09 |
|  | Spinal osteochondrosis | 0.99 (0.72, 1.36) | 0.96 | 1.11 (0.60, 2.05) | 0.74 | -0.055 | 0.55 | 18 | 0.65 |
|  | Intracranial and intraspinal abscess | 1.10 (0.76, 1.58) | 0.62 | 0.61 (0.30, 1.21) | 0.17 | 0.175 | 0.10 | 22 | 0.42 |
|  | Cervical spinal cord and nerve injuries | 0.95 (0.73, 1.23) | 0.69 | 0.96 (0.56, 1.66) | 0.89 | -0.005 | 0.95 | 22 | 0.42 |
|  |  |  |  |  |  |  |  |  |  |
|  | Glioblastoma | 0.95 (0.62, 1.46) | 0.83 | 0.83 (0.35, 1.97) | 0.67 | 0.063 | 0.63 | 16 | 0.75 |
|  | Benign meningioma | 1.01 (0.90, 1.14) | 0.85 | 1.08 (0.85, 1.39) | 0.53 | -0.036 | 0.33 | 13 | 0.92 |
|  | Malignant meningioma | 0.99 (0.84, 1.17) | 0.92 | 1.33 (0.96, 1.85) | 0.11 | -0.085 | 0.09 | 19 | 0.58 |
|  | Pituitary adenoma and craniopharyngioma | 1.01 (0.87, 1.18) | 0.88 | 0.91 (0.67, 1.23) | 0.54 | 0.043 | 0.36 | 18 | 0.66 |
|  | Benign neoplasm of brain and other parts of CNS | 0.96 (0.83, 1.11) | 0.57 | 1.05 (0.80, 1.39) | 0.72 | -0.020 | 0.62 | 18 | 0.67 |
|  | Malignant neoplasm of brain and other parts of CNS | 0.82 (0.62, 1.10) | 0.19 | 0.89 (0.49, 1.61) | 0.70 | 0.022 | 0.80 | 19 | 0.60 |
|  |  |  |  |  |  |  |  |  |  |
|  | Hydrocephalus | 1.06 (0.91, 1.23) | 0.47 | 1.26 (0.93, 1.72) | 0.15 | -0.070 | 0.14 | 18 | 0.65 |
|  | Craniosynostosis | 0.89 (0.72, 1.11) | 0.30 | 0.99 (0.65, 1.49) | 0.95 | 0.001 | 0.99 | 17 | 0.71 |
|  | Concussion | 1.01 (0.97, 1.06) | 0.60 | 1.03 (0.94, 1.13) | 0.52 | -0.005 | 0.72 | 23 | 0.36 |
|  | Diffuse brain injury | 1.21 (1.01, 1.45) | 0.04 | 1.48 (0.97, 2.26) | 0.08 | -0.091 | 0.16 | 37 | 0.02 |
|  | Focal brain injury | 1.02 (0.90, 1.15) | 0.79 | 1.11(0.86, 1.43) | 0.44 | -0.017 | 0.66 | 19 | 0.58 |
|  | Congenital malformations of the nervous system | 1.27 (0.99, 1.63) | 0.06 | 1.53 (0.91, 2.58) | 0.13 | -0.091 | 0.25 | 17 | 0.72 |

**Table S2** Associations between genetically predicted critically ill COVID-19 and 30 neurosurgical disorders in sensitivity analyses using the weighted-median and MR-Egger methods.
