# Supplementary material for: Causal associations of COVID‐19 on neurosurgical diseases risk: a Mendelian randomization study
Source: Hum Genomics. 2024 Feb 5;18:13. doi: 10.1186/s40246-024-00575-y (PMC10840232; doi:10.1186/s40246-024-00575-y)
Supplement: Supplementary file 3 — Additional file 3: Table S3. Associations between genetically predicted hospitalized COVID-19 and 30 neurosurgical disorders in sensitivity analyses using the weighted-median and MR-Egger methods. [file 40246_2024_575_MOESM3_ESM.docx]

| Outcome | | Weighted Median | | MR-Egger | | Pleiotropy | | Heterogeneity | |
| --- | --- | --- | --- | --- | --- | --- | --- | --- | --- |
|  |  | OR (95% CI) | P | OR (95% CI) | P | Intercept | P | Q | P |
| hospitalized COVID‐19 | Trigeminal neuralgia | 1.02 (0.90, 1.16) | 0.74 | 1.05 (0.90, 1.23) | 0.51 | -0.018 | 0.66 | 22 | 0.73 |
|  | Epilepsy | 1.02 (0.97, 1.07) | 0.40 | 0.99 (0.92, 1.07) | 0.82 | 0.004 | 0.79 | 33 | 0.32 |
|  | Parkinson's disease | 1.01 (0.95, 1.08) | 0.65 | 1.06 (0.97, 1.17) | 0.21 | -0.016 | 0.46 | 22 | 0.87 |
|  | Alzheimer's disease | 1.00 (0.98, 1.02) | 0.67 | 1.00 (0.97, 1.03) | 0.89 | 0.000 | 0.95 | 21 | 0.79 |
|  | Major depressive disorder | 1.01 (0.93, 1.10) | 0.74 | 1.17 (0.89, 1.53) | 0.27 | -0.041 | 0.35 | 11 | 0.98 |
|  | Obsessive Compulsive Disorder | 1.04 (0.94, 1.15) | 0.41 | 1.02 (0.84, 1.24) | 0.85 | -0.003 | 0.95 | 30 | 0.11 |
|  |  |  |  |  |  |  |  |  |  |
|  | Stroke | 1.00 (0.97. 1.03) | 0.97 | 1.00 (0.94. 1.07) | 0.98 | 0.011 | 0.80 | 14 | 0.53 |
|  | Intracerebral hemorrhage | 0.97 (0.91, 1.04) | 0.39 | 1.04 (0.94, 1.16) | 0.44 | -0.031 | 0.18 | 26 | 0.66 |
|  | Subarachnoid hemorrhage | 1.01 (0.94, 1.09) | 0.72 | 0.96 (0.86, 1.07) | 0.44 | 0.012 | 0.62 | 25 | 0.74 |
|  | Transient ischemic attack | 1.01 (0.97, 1.05) | 0.66 | 0.99 (0.94, 1.04) | 0.69 | 0.003 | 0.81 | 21 | 0.79 |
|  | Cerebral infarction | 1.00 (0.99. 1.01) | 0..97 | 1.00 (0.99. 1.01) | 0.57 | 1.79e-06 | 0.99 | 28 | 0.55 |
|  | Cerebral aneurysm | 0.99 (0.91, 1.08) | 0.85 | 1.00 (0.87, 1.15) | 0.96 | -0.008 | 0.77 | 23 | 0.82 |
|  |  |  |  |  |  |  |  |  |  |
|  | Cervical spondylosis | 1.00 (0.99, 1.01) | 0.21 | 1.00 (0.99, 1.01) | 0.69 | 0.000 | 0.32 | 31 | 0.33 |
|  | Spinal canal stenosis | 0.98 (0.94, 1.02) | 0.25 | 1.00 (0.94, 1.05) | 0.86 | -0.007 | 0.61 | 34 | 0.29 |
|  | spinal meningioma | 0.92 (0.65, 1.29) | 0.63 | 0.85 (0.55, 1.30) | 0.46 | -0.009 | 0.93 | 29 | 0.34 |
|  | Spinal osteochondrosis | 0.91 (0.71, 1.18) | 0.49 | 0.79 (0.57, 1.10) | 0.17 | 0.146 | 0.09 | 24 | 0.66 |
|  | Intracranial and intraspinal abscess | 1.26 (0.94, 1.68) | 0.13 | 0.89 (0.58, 1.36) | 0.60 | 0.102 | 0.35 | 35 | 0.14 |
|  | Cervical spinal cord and nerve injuries | 0.96 (0.78, 1.20) | 0.75 | 1.01 (0.77, 1.34) | 0.93 | -0.022 | 0.76 | 24 | 0.62 |
|  |  |  |  |  |  |  |  |  |  |
|  | Glioblastoma | 1.08 (0.74, 1.56) | 0.69 | 1.60 (1.00, 2.54) | 0.06 | -0.228 | 0.06 | 24 | 0.61 |
|  | Benign meningioma | 0.98 (0.88, 1.08) | 0.64 | 0.93 (0.81, 1.06) | 0.28 | 0.041 | 0.23 | 17 | 0.94 |
|  | Malignant meningioma | 0.96 (0.84, 1.10) | 0.52 | 0.92 (0.76, 1.11) | 0.37 | 0.024 | 0.62 | 31 | 0.27 |
|  | Pituitary adenoma and craniopharyngioma | 0.84 (0.74, 0.95) | 0.01 | 0.82 (0.68,0.99) | 0.05 | 0.052 | 0.29 | 37 | 0.10 |
|  | Benign neoplasm of brain and other parts of CNS | 0.95 (0.84, 1.07) | 0.38 | 0.94 (0.81, 1.09) | 0.40 | 0.040 | 0.31 | 29 | 0.37 |
|  | Malignant neoplasm of brain and other parts of CNS | 1.05 (0.81, 1.37) | 0.69 | 1.09 (0.76, 1.55) | 0.65 | -0.015 | 0.87 | 34 | 0.17 |
|  |  |  |  |  |  |  |  |  |  |
|  | Hydrocephalus | 0.96 (0.84, 1.09) | 0.52 | 1.11 (0.93, 1.32) | 0.26 | -0.058 | 0.20 | 32 | 0.23 |
|  | Craniosynostosis | 1.05 (0.89, 1.23) | 0.60 | 1.13 (0.91, 1.41) | 0.27 | -0.061 | 0.27 | 20 | 0.85 |
|  | Concussion | 1.02 (0.98, 1.06) | 0.33 | 0.99 (0.94, 1.04) | 0.64 | 0.012 | 0.31 | 28 | 0.41 |
|  | Diffuse brain injury | 1.09 (0.94, 1.26) | 0.24 | 0.92 (0.75, 1.12) | 0.43 | 0.083 | 0.11 | 38 | 0.07 |
|  | Focal brain injury | 1.12 (1.01, 1.25) | 0.04 | 1.09 (0.93, 1.27) | 0.30 | -0.002 | 0.96 | 35 | 0.15 |
|  | Congenital malformations of the nervous system | 1.00 (0.79, 1.26) | 0.98 | 1.02 (0.74, 1.42) | 0.90 | 0.006 | 0.94 | 37 | 0.10 |

**Table S3** Associations between genetically predicted hospitalized COVID-19 and 30 neurosurgical disorders in sensitivity analyses using the weighted-median and MR-Egger methods.
