# Supplementary material for: Causal associations of COVID‐19 on neurosurgical diseases risk: a Mendelian randomization study
Source: Hum Genomics. 2024 Feb 5;18:13. doi: 10.1186/s40246-024-00575-y (PMC10840232; doi:10.1186/s40246-024-00575-y)
Supplement: Supplementary file 4 — Additional file 4: Table S4. Associations between genetically predicted SARS‐CoV‐2 infection and 30 neurosurgical disorders in sensitivity analyses using the weighted-median and MR-Egger methods. [file 40246_2024_575_MOESM4_ESM.docx]

| Outcome | | Weighted Median | | MR-Egger | | Pleiotropy | | Heterogeneity | |
| --- | --- | --- | --- | --- | --- | --- | --- | --- | --- |
|  |  | OR (95% CI) | P | OR (95% CI) | P | Intercept | P | Q | P |
| SARS‐CoV‐2 infection | Trigeminal neuralgia | 1.09 (0.96, 1.24) | 0.20 | 1.02 (0.85, 1.24) | 0.81 | 0.008 | 0.75 | 24 | 0.98 |
|  | Epilepsy | 1.02 (0.96, 1.07) | 0.57 | 1.11 (1.02, 1.20) | 0.02 | -0.030 | 3.4e-03 | 55 | 0.10 |
|  | Parkinson's disease | 0.98 (0.91, 1.05) | 0.53 | 0.99 (0.89, 1.11) | 0.88 | -0.002 | 0.88 | 35 | 0.80 |
|  | Alzheimer's disease | 1.00 (0.98, 1.02) | 0.95 | 0.99 (0.95, 1.03) | 0.52 | 0.004 | 0.37 | 57 | 0.06 |
|  | Major depressive disorder | 1.04 (0.96, 1.12) | 0.34 | 1.09 (0.84, 1.41) | 0.53 | -0.012 | 0.61 | 20 | 0.99 |
|  | Obsessive Compulsive Disorder | 1.01 (0.91, 1.11) | 0.92 | 1.09 (0.89, 1.34) | 0.40 | -0.015 | 0.50 | 24 | 0.96 |
|  |  |  |  |  |  |  |  |  |  |
|  | Stroke | 1.03 (1.00, 1.05) | 0.03 | 1.02 (0.98, 1.07) | 0.35 | -0.001 | 0.83 | 48 | 0.13 |
|  | Intracerebral hemorrhage | 1.03 (0.96, 1.10) | 0.46 | 0.99 (0.87, 1.12) | 0.84 | -0.002 | 0.86 | 47 | 0.31 |
|  | Subarachnoid hemorrhage | 1.02 (0.94, 1.10) | 0.70 | 1.09 (0.95, 1.24) | 0.25 | -0.020 | 0.20 | 50 | 0.21 |
|  | Transient ischemic attack | 1.00 (0.95, 1.05) | 0.97 | 1.00 (0.94, 1.06) | 0.95 | 0.002 | 0.85 | 38 | 0.57 |
|  | Cerebral infarction | 1.00 (0.99, 1.01) | 0.21 | 1.00 (0.99, 1.01) | 0.49 | -1.49e-04 | 0.14 | 44 | 0.38 |
|  | Cerebral aneurysm | 0.98 (0.91, 1.05) | 0..58 | 0.93 (0.81, 1.08) | 0.35 | 0.011 | 0.47 | 27 | 0.98 |
|  |  |  |  |  |  |  |  |  |  |
|  | Cervical spondylosis | 1.00 (0.99, 1.01) | 0.65 | 1.00 (0.99, 1.01) | 0.79 | -1.45e-05 | 0.87 | 44 | 0.41 |
|  | Spinal canal stenosis | 1.04 (1.00, 1.08) | 0.06 | 1.05 (0.99, 1.11) | 0.14 | -0.004 | 0.57 | 40 | 0.62 |
|  | spinal meningioma | 1.15 (0.81, 1.64) | 0.44 | 1.48 (0.89, 2.47) | 0.14 | -0.057 | 0.38 | 43 | 0.35 |
|  | Spinal osteochondrosis | 0.95 (0.72, 1.26) | 0.72 | 1.02 (0.66, 1.58) | 0.92 | -0.020 | 0.72 | 48 | 0.19 |
|  | Intracranial and intraspinal abscess | 1.17 (0.86, 1.60) | 0.31 | 1.02 (0.65, 1.59) | 0.94 | 0.033 | 0.56 | 36 | 0.64 |
|  | Cervical spinal cord and nerve injuries | 0.89 (0.70, 1.12) | 0.32 | 0.96 (0.69, 1.35) | 0.82 | 0.001 | 0.97 | 31 | 0.83 |
|  |  |  |  |  |  |  |  |  |  |
|  | Glioblastoma | 0.80 (0.53, 1.19) | 0.27 | 0.81 (0.44, 1.49) | 0.51 | 0.035 | 0.65 | 47 | 0.22 |
|  | Benign meningioma | 1.01 (0.90, 1.13) | 0.85 | 1.09 (0.92, 1.29) | 0.32 | -0.022 | 0.30 | 44 | 0.32 |
|  | Malignant meningioma | 1.05 (0.90, 1.23) | 0.53 | 1.11 (0.89, 1.38) | 0.36 | -0.025 | 0.37 | 42 | 0.37 |
|  | Pituitary adenoma and craniopharyngioma | 1.03 (0.90, 1.19) | 0.63 | 0.99 (0.81, 1.21) | 0.96 | 0.009 | 0.73 | 40 | 0.46 |
|  | Benign neoplasm of brain and other parts of CNS | 0.96 (0.85, 1.09) | 0.53 | 1.18 (0.99, 1.41) | 0.08 | -0.045 | 0.05 | 37 | 0.59 |
|  | Malignant neoplasm of brain and other parts of CNS | 1.17 (0.88, 1.57) | 0.28 | 1.57 (1.02, 2.41) | 0.05 | -0.076 | 0.17 | 52 | 0.09 |
|  |  |  |  |  |  |  |  |  |  |
|  | Hydrocephalus | 0.97 (0.85, 1.12) | 0.70 | 0.94 (0.76, 1.17) | 0.60 | 0.023 | 0.38 | 45 | 0.26 |
|  | Craniosynostosis | 0.91 (0.75, 1.10) | 0.34 | 1.09 (0.82, 1.46) | 0.55 | -0.038 | 0.29 | 47 | 0.21 |
|  | Concussion | 0.99 (0.95, 1.03) | 0.56 | 0.97 (0.91, 1.02) | 0.24 | 0.007 | 0.34 | 42 | 0.37 |
|  | Diffuse brain injury | 0.98 (0.84, 1.14) | 0.76 | 1.04 (0.84, 1.29) | 0.69 | -0.022 | 0.41 | 41 | 0.42 |
|  | Focal brain injury | 0.96 (0.86, 1.08) | 0.52 | 0.95 (0.80, 1.12) | 0.52 | 0.011 | 0.60 | 31 | 0.86 |
|  | Congenital malformations of the nervous system | 1.13 (0.88, 1.47) | 0.34 | 1.28 (0.88, 1.87) | 0.20 | -0.039 | 0.41 | 50 | 0.14 |

**Table S4** Associations between genetically predicted SARS‐CoV‐2 infection and 30 neurosurgical disorders in sensitivity analyses using the weighted-median and MR-Egger methods.
